# Phylogenetics of the *Antopocerus*-*Modified Tarsus* Clade of Hawaiian *Drosophila*: Diversification across the Hawaiian Islands

**DOI:** 10.1371/journal.pone.0113227

**Published:** 2014-11-24

**Authors:** Richard T. Lapoint, Karl N. Magnacca, Patrick M. O’Grady

**Affiliations:** 1 Department of Ecology and Evolutionary Biology, University of Arizona, Tucson, AZ, United States of America; 2 Oahu Army Natural Resource Program, Honolulu, HI, United States of America; 3 Department of Environmental Science, Policy and Management, University of California, Berkeley, CA, United States of America; University of Arkansas, United States of America

## Abstract

The Hawaiian Drosophilidae radiation is an ecologically and morphologically diverse clade of almost 700 described species. A phylogenetic approach is key to understanding the evolutionary forces that have given rise to this diverse lineage. Here we infer the phylogeny for the *antopocerus*, *modified tarsus* and *ciliated tarsus* (AMC) clade, a lineage comprising 16% (91 of 687 species) of the described Hawaiian Drosophilidae. To improve on previous analyses we constructed the largest dataset to date for the AMC, including a matrix of 15 genes for 68 species. Results strongly support most of the morphologically defined species groups as monophyletic. We explore the correlation of increased diversity in biogeography, sexual selection and ecology on the present day diversity seen in this lineage using a combination of dating methods, rearing records, and distributional data. Molecular dating analyses indicate that AMC lineage started diversifying about 4.4 million years ago, culminating in the present day AMC diversity. We do not find evidence that ecological speciation or sexual selection played a part in generating this diversity, but given the limited number of described larval substrates and secondary sexual characters analyzed we can not rule these factors out entirely. An increased rate of diversification in the AMC is found to overlap with the emergence of multiple islands in the current chain of high islands, specifically Oahu and Kauai.

## Introduction

### Diversity in the Hawaiian Islands

The extreme isolation and varied ecological habitats present in the Hawaiian Islands makes this archipelago home to high levels of endemism [1] and a model system for studying diversification. Many large radiations are known from the Hawaiian Islands, with well-known examples from plants [2], [3], vertebrates [4], and invertebrates [5]–[8]. Price and Clague [9] reviewed Hawaiian lineages with estimated colonization and divergence dates and found that most groups arrived in Hawaii immediately following the formation of the current high islands, approximately 5.2 million years ago (mya). Given its scope and relative recency, understanding what influenced the current diversity in the Hawaiian Islands is a challenge.

**Figure 1:**
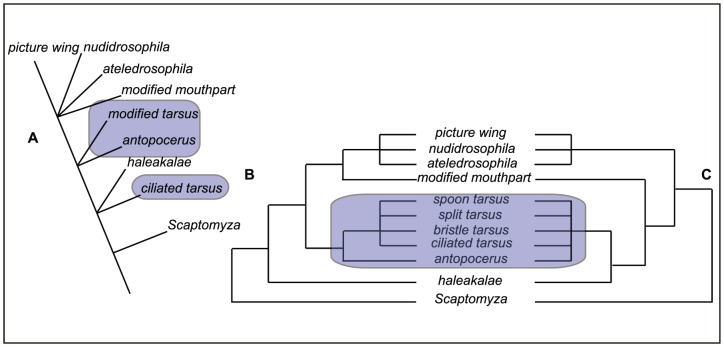
Previous phylogenetic hypotheses regarding relationships within the Hawaiian *Drosophila*. Highlighted boxes include the placement of the AMC clade. A) Relationships based on internal morphology [32]; B) combined nuclear and mitochondrial gene sequences for 9 representative AMC species [38]; C) Mitochondrial sequence data for 55 AMC species [7].

Only five endemic lineages are inferred to have colonized the Hawaiian archipelago prior to the formation of the current high islands based on current dated phylogenies [10]–[14]. The oldest and most diverse of these is the Hawaiian Drosophilidae, a radiation derived from a common ancestor approximately 25 mya [10], [15], [16]. The Drosophilidae endemic to Hawaii have diversified into two main clades, the Hawaiian *Drosophila*, or *Idiomyia* [17], and the genus *Scaptomyza* (Figure 1). Combined, this lineage contains an estimated 1000 species, of which 687 are currently described [18]. The Hawaiian Drosophilidae are well known for their ecological diversification in larval host use and lekking sites [19]–[23] as well as their remarkable degree of morphological variation [24], [25]. A number of forces, including ecological adaptation [22], [23], sexual selection [26] and allopatric speciation [27], have been implicated in generating the current high level of species diversity in the Hawaiian Drosophilidae.

### The AMC as a model for Hawaiian diversity

This study focuses on the *antopocerus*, *modified tarsus*, and *ciliated tarsus* (AMC) clade, a radiation of 91 described species of Hawaiian *Drosophila*, to explore the factors that may have produced its current diversity. The AMC taxa are placed in two lineages, the *antopocerus* species group, which includes the *adunca*, *diamphidiopoda* and *villosa* subgroups, and the *modified tarsus* species group, *sensu* O’Grady et al. [18], containing the *bristle tarsus*, *ciliated tarsus*, *split tarsus*, and *spoon tarsus* subgroups. The morphological diversity found in species of the AMC clade is striking. The *antopocerus* species group, once considered to belong to a different genus, is comprised of large flies (up to 6 mm long) with long whip-like antennae [28]. The species placed in the *modified tarsus* species group are defined by secondary sexual characters on the foretarsi of the males which include reduced numbers of segments, spoon-shaped structures, elongate cilia, or thick clusters of setae [24], [29]–[31]. These tarsal modifications are used in mating displays and behaviors [32]. Despite the extensive morphological diversity in this lineage, the majority of the AMC clade with known ecological affinities utilize decaying leaf matter as their primary oviposition substrate, a behavior that has caused some authors [19], [33] to refer to these species as the “leaf breeders.” In addition to oviposition preference, members of this lineage share similar male genitalia, internal anatomy and mating and lekking behaviors [28], [32], [34]–[37].

Our understanding of the phylogenetic relationships within species groups, subgroups and lineages of the AMC clade, as well as its placement within the Hawaiian *Drosophila* has changed over the past four decades (Fig. 1). The earliest morphological analysis united the *antopocerus* species group and the *modified tarsus* species group, and placed them sister to the *modified mouthpart* and *picture wing* clades, with the *ciliated tarsus* species separate (Fig. 1A; [33]). Molecular phylogenies have solid support for the monophyly of the AMC clade (Fig. 1B, Fig. 1C), but lack either enough characters or taxa to adequately resolve relationships within the group with support. A number of studies analyzed representatives from one or two AMC species subgroups [23], [38], [39], but until recently did not include enough exemplars for a rigorous assessment of the monophyly within the AMC or relationships between the species groups therein. In the largest analysis to include the AMC, O’Grady et al. [7] used four mitochondrial loci to infer the phylogeny of the Hawaiian Drosophilidae. This analysis included 54 AMC species, including multiple representatives from all the major species groups and subgroups of this clade. The monophyly of the AMC clade as a whole, as well as several lineages (*e.g., spoon tarsus, antopocerus*), were well supported. Support for relationships between the AMC species groups were not strong, perhaps owing to the limited number of loci used in this study.

### Objectives

Here we improve on previous phylogenetic studies to clarify the relationships within the AMC clade and test the factors that may have driven its diversification. The current study includes 68 AMC species, the largest number sampled to date and over 75% of the described species diversity, as well as representative taxa from all Hawaiian *Drosophila* species groups for outgroups. To assemble the largest sequence matrix to date for this lineage we include sequence data from 10 nuclear and five mitochondrial loci. We use these data to estimate divergence times in the AMC clade and attempt to correlate rate and timing of diversification events within the AMC with various factors implicated in generating diversity in the Hawaiian *Drosophila* and other Hawaiian arthropod groups. The factors we test include the importance of the Hawaiian Island geography, specifically the progression rule [40], [41], habitat availability [4], ecological opportunity [42], and the increase in diversity of sexually selected characters [43].

## Methods

### Sampling, DNA amplification and sequencing

Sixty-eight AMC clade species were collected from localities across the current Hawaiian high islands (which include Hawaii, Maui, Molokai, Oahu, and Kauai) (Table S1). Specimens from all five AMC lineages (*antopocerus* species group, *split tarsus*, *spoon tarsus*, *ciliated tarsus* and *bristle tarsus* subgroups) were included. Flies were swept from the leaf litter or aspirated directly from sponges soaked with fermenting banana or mushroom baits. Permits for collecting Hawaiian *Drosophila* were issued from the Hawaii Natural Area Reserves, Department of Fish and Wildlife, and Hawaii Volcanoes National Park. Specimens were stored in 95% alcohol for subsequent identification and DNA extraction. Species identifications were performed by the authors using published keys [28]–[31]. Of the 68 AMC taxa we sampled, 59 were identified to species, comprising 64.8% of the known diversity of this clade [18]. Another nine specimens described as “near” a described species (sp. nr.) were included in the analyses. While these specimens were clearly closely allied with described species, they did not conform to the morphological concept of the known species, and await description. Based on the number of sp. nr. taxa collected in the past 10 years, we predict that there may be more AMC species awaiting discovery and description. In spite of the potential for undescribed species in the Hawaiian fauna, we feel our sampling represents a significant portion of the known diversity and is representative of the extant beta diversity at the level of species group and subgroup in the AMC clade.

Outgroup taxa were selected to test the monophyly of the AMC clade and to facilitate dating analyses. Outgroups were chosen from across the three other Hawaiian *Drosophila* clades [7]: *haleakalae* species group (*D ochropleura*), *modified mouthpart* species group (*D. nigrocirrus*), *picture wing* clade (*D. grimshawi*) and from the genus *Scaptomyza* (*S. varipicta*), the sister lineage of the Hawaiian *Drosophila*.

Genomic DNA was extracted from individual flies using the Qiagen DNeasy DNA extraction kit (Qiagen, Inc). The only departure from the standard protocol was that whole individuals of rarely collected species were soaked in Proteinase K and subsequently preserved as point mounted vouchers. Individuals were macerated according to manufacturers protocol only when a series of conspecifics from the same locality and date were available to preserve as vouchers in 95% alcohol. All voucher material has been deposited into the B.P. Bishop Museum as pinned material or remains as an ethanol voucher at the Essig Museum of Entomology at UC Berkeley. For details on individuals contact the authors with the appropriate barcodes from Table S1.

We sequenced a panel of 10 nuclear loci and five mitochondrial genes to infer phylogenetic relationships among the AMC individuals in this study. The mitochondrial loci *nd2*, *nd4*, *co1*, *co2*, and *16s* were amplified using universal mitochondrial primers [44]. The nuclear loci included were *fz4*, *kl2*, *pds5* [45] *snf, wee, ntid*, *boss* [46], [47], *yp1*, *yp2* [23] and *ef1g* (Table 1). PCR products were cleaned using a standard ExoSAP-IT protocol (USB). Cleaned products were sequenced in both directions on an ABI 3730 capillary sequencer. Contigs were assembled using Sequencher, ver. 4.7 (GeneCodes, Corp). Newly generated sequences were deposited in Genbank. When available, additional sequences were downloaded from Genbank for conspecific taxa (Table S1 and S2). Sequence divergence between species was low and alignment was trivial. Sequences were aligned to the orthologous *D. grimshawi* sequence in MacClade, ver. 4.06 [48] using the default parameters in the Needleman-Wunsch algorithm [49] included in this software package. Sequence alignments for protein coding loci were translated to improve gap placement, and misalignments were identified when coding regions were not in frame and corrected manually. Six to 15 genes were sequenced for all taxa, with each species having an average of 11.2 genes sequenced. The concatenated matrix is 67% complete and taxon coverage for each gene matrix ranges from 31% of sampled taxa (*yp2*) to 99% of sampled taxa (*co2*). The lowest taxa coverage per gene was for the genes *yp1*, *yp2*, *ef1g* and *nd4.* The 15 aligned genes comprised a matrix of 9884 base pairs, which includes gaps and unknown bases (Table 1).

**Table 1:** Gene details and diversity.

Locus	Primer Label	Primer Sequence (5′–3′)	Locus Type	Aligned Length	PIC^a^	Individuals Sequenced
Kl-2	Kl2L	TAATACAGAACGGTGGTATGGGTAT	Y	571	63	60
	kl2R	GTTGCTTGGCTAATTCGTAAAGAGT				
Fz4	Fz4L	GCGTCTTTCTATTGCGCTACTAT	X	974	89	55
	Fz4R	GCTTGTACGGACTGCTGATTATT				
Snf	snfL	GAAGATGCGGGGCCARGCNTTYGT	X	395	81	69
	snfR	GAACAGCATGGACAGCATCATYTCRTT				
Yp1	YP1D-F	GGACAGGATGAGGTNACCATCATTGT	X	911	50	23
	YP1D-R	TGRTAGCTGTTCTGCTTCTGGGC				
Yp2	YP2F	CAGCAGCGTTACAATCTCCAGCC	X	688	25	22
	YP2R	CCGAAGGGGCTCTTGGAGTTCAC				
Pds5	Pds5L	GGATACTTTGTGGACAATTCAGAGT	autosomal	594	87	64
	Pds5R	AGATATTTCACGAACTCTTCAGCAC				
Boss	BossF1	ACCAGATGCCCTGGGGNGARAA	autosomal	726	136	53
	BossR1	TGGACAGGGAGCCGCKNARCCARTT				
Ntid	ntidF1	GGGCCGCATCTTCGARCAYAARTGG	autosomal	567	98	69
	ntidR1	TGGAGGGGTAGGTGTTCCARCARTA				
Wee	weeL	GCCTGGGCCGAGGAYGAYCAYATG	autosomal	297	40	56
	weeR	TCACGTGGCCCAGGTCNCCDATYTT				
Ef1g	EF1g26F	GCTTWTGAGACCGCTGATGG	autosomal	844	20	23
	EF1g862R	ATCTTRTCGAGACGCTGGAA				
ND2	192	AGCTATTGGGTTCAGACCCC	mito	523	132	69
	732	GAAGTTTGGTTTAAACCTCC				
ND4	FN4F	GATACAGGAGCTTCTACATGAGC	mito	687	79	23
	FN4R	GTTTGTGAAGGAGCATTAGG				
COI	2183	CAACATTTATTTTGATTTTTTGG	mito	831	201	71
	3037	TYCATTGCACTAATCTGCCATATTAG				
COII	3041	ATGGCAGATTAGTGCAATGG	mito	765	167	70
	3791	GTTTAAGAGACCAGTACTTG				
16s	16sF	CCGGTTTGAACTCAGATCACGT	mito	511	27	69
	16sR	CGCCTGTTTAACAAAAACAT				
Total				9884	1295	

a) Number of parsimony informative characters.

### Phylogenetic Inference

Individual gene genealogies were estimated using Bayesian [50] and maximum likelihood inference methods [51]. Datasets were partitioned by codon positions (1^st^, 2^nd^, 3^rd^), non-coding regions (introns), and structural rRNA. The best-fit substitution model for each partition in the Bayesian analyses was estimated via the Akaike information criterion (AIC), implemented in MrModeltest, ver. 2.3 (Table S4) [52]. Individual Bayesian gene phylogenies were inferred by running the analyses for one million generations, with sampling every 100 generations. We examined the cumulative split frequencies plot calculated by AWTY [53] and identified when the potential scale reduction factor (PSRF) approached 1 [54] to assess the convergence of the Bayesian analyses. Appropriate levels of burn-in were discarded – generally the first 10% to 20% of the sampled data. The GTRGAMMA model was applied to each partition in the likelihood analysis [51]. Five hundred bootstrap replicates were performed to assess support for the inferred relationships. Each dataset was analyzed five times with different random starting seeds in RAxML to identify if significant changes in topology and support occurred between runs. Both the Bayesian and maximum likelihood analyses were performed on XSEDE, accessed through the CIPRES portal [55].

The entire dataset of 15 loci was concatenated and partitioned by gene and codon position. This dataset was analyzed in both maximum likelihood [51] and Bayesian [56] frameworks. We included all partitions from the genealogical analyses for a total of 49 partitions. The GTRGAMMA was applied to all partitions in the likelihood analysis. The concatenated dataset was analyzed ten times with different random starting seeds in RAxML to identify if significant changes in topology and support occurred between runs. One thousand bootstrap replicates were performed to assess support for the inferred relationships.

BEAST v1.8.0 was used to simultaneously infer the topology and age of nodes in the concatenated partitioned analysis [56]. An uncorrelated lognormal model of rate variation and a birth-death speciation process for branching rates was used. The analysis was run four times for 100 million generations, sampling every 10000 generations. Output files were combined using LogCombiner v1.8.0. Tracer v1.6 [57] was used to assess convergence and to identify if the posterior distribution of all parameters had an effective sample size (ESS) of>200 and were therefore adequately sampled.

Three nodes were calibrated using probabilistic priors. The oldest calibration point is the split between *Scaptomyza* and the Hawaiian *Drosophila*. This node was calibrated using a uniform prior ranging from 23.9 to 37.1 mya based on the range of ages of this split inferred by prior studies [10], [15], [16]. We also used the geologic history of the Hawaiian Islands to inform two node ages. Clades endemic to the island of Hawaii are not expected to be older than that island [57], making the most probable time of divergence between Hawaiian endemic lineages and their sister species on the next nearest island, Maui, about 0.59 mya [4], [58]. Since the lineage endemic to the island of Hawaii could have diverged before the formation of Hawaii and sister species on Maui Nui went extinct, or the island of Hawaii could have been colonized later than the island's initial formation, we calibrated the time to most recent common ancestor of these groups with a normal distribution prior with a mean of 0.59 and a standard deviation of 0.135 mya. This creates a distribution where the most probable time of divergence is 0.59 mya, but allows for divergence from almost the present and up to 0.9 mya [59]. The Hawaii Island endemic clades of *spoon tarsus* and *antopocerus* species were calibrated using this prior.

We tested the dated phylogeny for changes in diversification rate to evaluate whether the AMC clade has undergone an increased rate of diversification. SymmeTREE v1.1 was used to identify if there has been a change in the diversification rate between lineages by comparing the amount of branching in the AMC tree to the expected amount of branching under a pure Yule model [60]. Rate shifts were evaluated using the Δ_1_ statistic under default conditions with the maximum clade credibility phylogeny obtained from the BEAST analysis with outgroups removed. A lineage through time plot was explored in LASER v2.3 with the pruned phylogeny. The γ statistic was calculated to identify if the rate of lineage accumulation is slowing compared to older bursts of speciation. Since incomplete taxon sampling is expected to simulate a slow down, we implement an MCCR method to test if the γ is still significant given the amount of missing species in the dataset [61]. The number of missing species was estimated after the list of described species from O’Grady et al. [18].

### Ancestral State Reconstructions

To infer the ancestral range of each lineage, species was coded as being from Hawaii, Maui Nui (including Maui, Molokai and Lanai), Oahu, or Kauai, or a combination thereof. Species from any of the four islands that make up Maui Nui were treated as being from one island. These islands were connected in the very recent past and these land bridges may have facilitated dispersal between volcanoes [62]. Despite the majority of Hawaiian *Drosophila* species being single island endemics (90%) [29], a large proportion (30.7%) of the species included in this study are found on one or more islands in Maui Nui. The range of each species in these analyses was coded after published collection records [28]–[31], or, in the case of undescribed species, our collection records. A dispersal, extinction and cladogenesis (DEC) model was implemented in the program Lagrange to infer the ancestral ranges of each species group [63]. Lagrange employs a likelihood framework to infer geographical range evolution on phylogenetic trees, while inferring rates of dispersal and local extinction. Since this analysis requires the phylogeny to be time calibrated we used the phylogeny inferred via BEAST with outgroup taxa pruned from the tree. The input file was formatted using the Lagrange configurator (www.reelab.net/lagrange/configurator). Dispersal was modeled to limit migration only between adjacent islands and, alternatively, to allow migration to occur between any island. We allowed for ancestral ranges to include multiple adjacent islands since several species are currently resident on adjacent islands.

Stochastic mapping (SM), a Bayesian method that can be applied to ancestral state reconstruction, was used to infer the ancestral states for host substrate and secondary sexual character states. This method infers the probability of a state change dependent on branch length and evolutionary rate and incorporates phylogenetic uncertainty into the reconstruction of the ancestral state. SM analysis was performed using SIMMAP 1.5 [64] on a sub-sample of 1000 trees from the posterior distribution of trees generated by BEAST. The overall substitution rate of each character was modeled using a gamma distribution whose priors α and β were estimated using the two-step procedure suggested in SIMMAP 1.5. Initially an MCMC analysis was used to sample overall rate parameter values. The results of this analysis were analyzed with the R Statistical Package and the sumprmcmc.r script provided with SIMMAP 1.5 to find the best fitting gamma and beta distributions. These priors were then included in a full ancestral state reconstruction analysis. Since diversification in host substrate use has been implicated as a contributor to the high rate of diversification in other Hawaiian *Drosophila* clades [7], [22], [23] we mapped larval host substrate onto our phylogeny. While not nearly as diverse as host use in other lineages, we wanted to explore this in the AMC as a comparison to the other Hawaiian *Drosophila* clades. Host substrate was identified based on known rearing records [21].

We also mapped secondary sexual characters onto the AMC phylogeny to test whether diversity in secondary sexual characters predates or antedates a high rate of diversification. The traits we used may be under sexual selection and are used in mating behaviors [32], [34]–[37]. We infer that sexual selection may drive diversification in the AMC if there is an increase in the diversity of these characters correlated with a change in diversification rate of the AMC and there is no evidence of ecological or biogeographical divergence. We coded four dimorphic male characters for species with adequate data: 1) the presence of a “split” tarsus, a state which describes a foreleg with an apical lobe on the basitarsus and a missing tarsal segment, 2) the presence of long, whip-like aristae, 3) the presence of “spoons,” or enlarged, concave structures present on the second tarsal segments on the forelegs, 4) the ratio of basitarsus length to the length of the setae on the forelegs. The continuous measures of the basitarsus to setae length were binned into 5 categories. Characters for each species were identified based on published species descriptions [28]–[31]. We mapped these characters in the same way as we did with the larval host substrate, using Simmap [64]. The appropriate gamma and beta distributions were estimated in the same way as described above.

## Results

### Phylogenetic Inference

**Figure 2:**
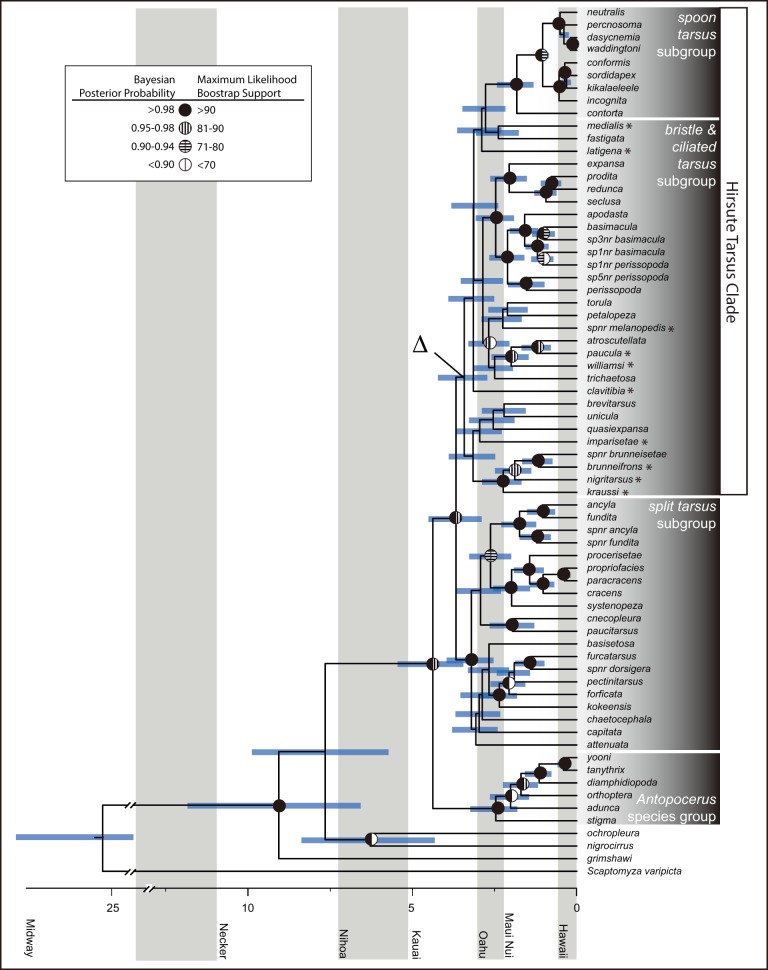
Chronogram of the AMC estimated in BEAST. Node bars indicate age range. Alternating grey and white bands indicate time when an island became aerial until next the island became aerial. Nodal support indicated at nodes as circles: Bayesian Posterior probabilities to the left and likelihood bootstrap values to the right. Nodes without circles have both measures of support less than 0.9 PP and 70% BS. Node of rate increase indicated by Δ. To delimit between species previously defined as belonging to either the *bristle* or *ciliated tarsus* species group we have identified *bristle tarsus* species with an asterisk next to their name, and all un-annotated species in the *bristle* and *ciliated tarsus* subgroup are *ciliated tarsus* species.

While individual gene phylogenies were poorly resolved (Fig. S1–S11), there was little conflict at nodes that had high support in all genealogies. We will restrict our discussion of relationships in this group to the partitioned, concatenated analyses that provide the greatest resolution between taxa and a marked improvement over previous work. The combined, concatenated analysis was an improvement over single gene phylogenies, with most nodes resolved with high statistical support in both the likelihood and Bayesian analyses (Fig. 2, Fig. S12). This phylogeny is also an improvement over previous molecular analyses of these groups, in terms of taxa inclusion and nodal support [7], [45]. The *antopocerus* species group, *split tarsus* subgroup and *spoon tarsus* subgroup were each monophyletic with a posterior probability (PP) of 1.0 and the lowest bootstrap support (BS) at 95% in the concatenated phylogeny (Fig. 2).

The *antopocerus* species group was inferred as monophyletic (BS = 100; PP = 1.0), an unsurprising result considering the substantial morphological differences between this species group and the *modified tarsus* species group [28]. Most species in the *antopocerus* species group are endemic to the islands of Maui Nui, with the exception of *D. cognata*, *D. kaneshiroi* (both not included in this analysis), *D. yooni* and *D. tanythrix* (all from Hawaii) and *D. arcuata* (Oahu, not included in these analyses). The species endemic to Hawaii were sister to each other (BS = 100; PP = 1.0) and nested within a paraphyletic grade of Maui endemics (Fig. 2).

The *antopocerus* species group was sister to the *modified tarsus* species group (BS = 80%, PP = 1.0). The *modified tarsus* species group is comprised of 75 species from the *spoon*, *bristle*, *ciliated* and *split tarsus* subgroups (Fig. 2). The *split tarsus* subgroup was estimated as the largest clade, with 24 described species found on all of the main Hawaiian Islands. Like the *antopocerus* species group, the *split tarsus* species were strongly supported as monophyletic in the concatenated analysis, and this clade was also found in some of the individual gene analyses (Fig. S1–S2, and S9–S10).

The split tarsus subgroup was well supported as the sister clade to what we are calling the *hirsute tarsus* clade (BS = 78; PP = 1.0), a group containing the *spoon tarsus*, *bristle tarsus*, and *ciliated tarsus* subgroups (BS = <50%, PP = 0.97). The *spoon tarsus* subgroup was monophyletic (BS = 100; PP = 1.0) and nested within a paraphyletic grade of *ciliated* and *bristle tarsus* species. The *spoon tarsus* species are united by possessing a distinctly cuplike structure with dense cilia within the concavity on the second tarsal segment of males [24]. While the *spoon tarsus* subgroup is described as containing 12 species [31], the validity of the morphological characters that have previously included *D. atroscutellata* and *D. fastigata* into this group has been questioned based on their indistinct “spoons” [24] and phylogenies containing these species have indicated that *D. fastigata* and *D. atroscutellata* may not be part of a monophyletic *spoon tarsus* grouping [7], [45]. Our analyses agreed with the previous phylogenetic work and indicated that the inclusion of *D. atroscutellata* and *D. fastigata* (included in this analysis) in the *spoon tarsus* subgroup was not warranted. This reconfigured *spoon tarsus* subgroup was strongly supported as monophyletic (BS = 99; PP = 1.0). This group was nested within a paraphyletic grade of *ciliated* and *bristle tarsus* species.

The remaining taxa placed in the hirsute tarsus clade, all members of the *bristle* and *ciliated tarsus* subgroups all share elongate setae on the forelegs of males. The *bristle tarsus* subgroup displays a clump of stiff bristles at the apex of the basitarsus, with some taxa also possessing an expanded or widened first tarsal segment. In contrast, the *ciliated tarsus* subgroup is characterized by more diffuse bristles along the tarsal segments on the forelegs of the males without any expansion or thickening of the tarsal segments. These two species groups were not reciprocally monophyletic. Instead, they comprised a paraphyletic grade of species including the monophyletic *spoon tarsus* subgroup. The chaetotatic characters used to define the *ciliated* and *bristle tarsus* species groups are not diagnostic taxonomically. This was not surprising given that elongate cilia on the forelegs of males are found throughout the Hawaiian Drosophilidae (e.g., [29]). Most of the *bristle tarsus* species that exhibit the most distinctive morphology, including a dorsal and an anterior row of sinuate, spinose setae, group into a single strongly-supported clade. These include *D. apodasta*, *D. basimacula*, *D. expansa*, and *D. perissopoda*. This clade includes other species that display a more poorly-defined bristling, such as *D. prodita*. However, two species with basitarsal bristle morphology virtually identical to that found in the group containing *D. apodasta* - *D. petalopeza* and *D. quasiexpansa* - fell out separate from both the main grouping and from each other, indicating strong convergent evolution in this trait.

### Dating and Rate of Diversification

The analysis converged quickly as indicated by an ESS well over 1000 for most parameters and greater than 500 for all others [56]. These analyses suggested that the AMC started diversifying about 4.4 mya, when the ancestors of the *antopocerus* and *modified tarsus* species group diverged (Fig. 2, Table 2). The *split tarsus* and the remainder of the *modified tarsus* taxa diverged about 3.3 mya (Fig. 2, Table 2). Divergence times within in the remainder of the AMC clade are listed in Table 2.

**Table 2:** Ages of Major Lineages.

Node	Node Age (95% HPD)
Hawaiian Drosophilidae	25.15 (23.90 – 27.46)
Hawaiian *Drosophila*	9.14 (6.57 – 11.82)
AMC	4.40 (3.45 – 5.45)
*antopocerus* species group	2.48 (1.81 – 3.24)
*modified tarsus* species group	3.69 (2.90 – 4.51)
*split tarsus* subgroup	3.22 (2.54 – 3.96)
*hirsute tarsus* subgroup	3.44 (2.72 – 4.22)
*spoon tarsus* subgroup	1.84 (1.32 – 2.43)

Ages of major lineages, species groups and subgroups estimated by BEAST. Important ages with 95% highest posterior densities. Refer to Figure 2 to identify nodes.

**Figure 3:**
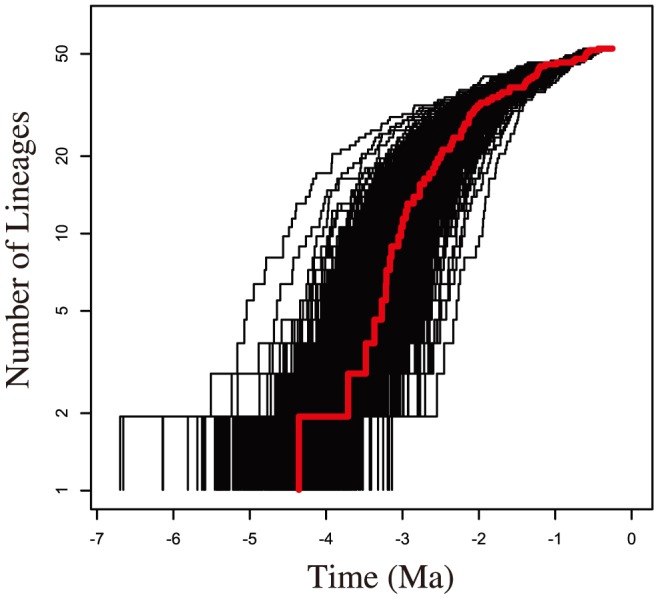
Lineage through time plot. Lineage through time plot of Maximum clade credibility tree in red. Lineage through time plot of 1000 post burn in topologies from the BEAST analysis in black.

SymmeTREE indicated a significant Δ_1_ (p = 0.01) increase in the rate of diversification at the base of the *hirsute tarsus* clade (Fig. 2). Additionally, the γ statistic was found to be significant (p = 0.001), suggesting that the rate of diversification has slowed closer to the tips of the tree from a high rate deeper in the topology. Lineage growth was qualitatively examined in the lineage through time plot (Fig. 3). The lineage through time plot displays an initially high rate of diversification at the base of the AMC phylogeny, that tapers off as time progresses. This pattern is predicted to indicate an adaptive radiation: The initial burst may be due to increased available resources, and the later slow down is expected to be caused by a decrease in available resources as more species compete over them [65].

### Ancestral State Reconstructions

**Figure 4:**
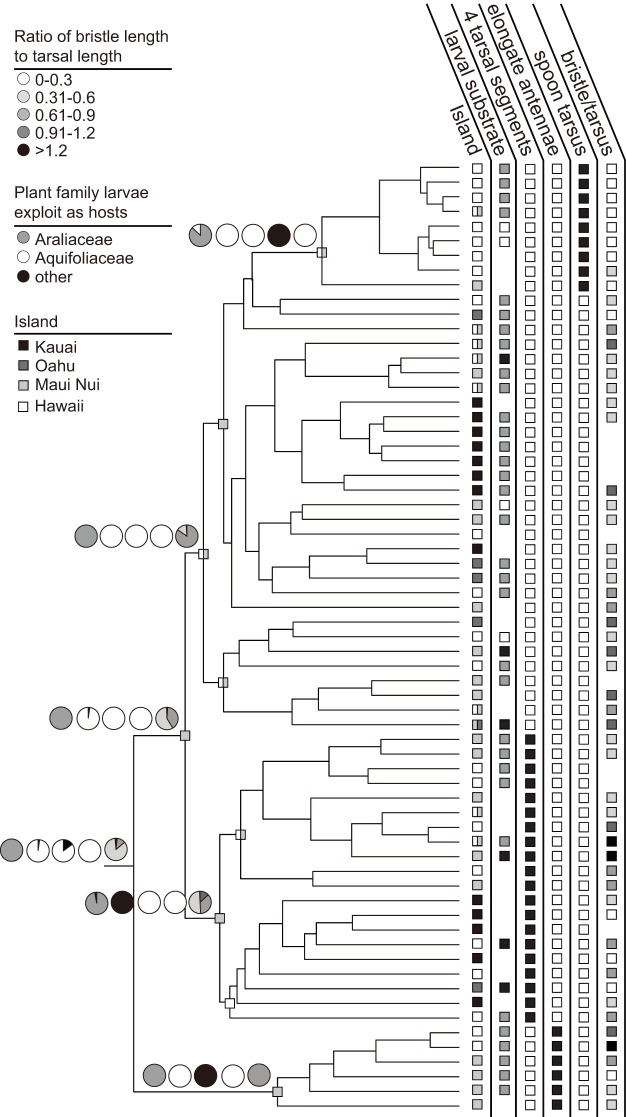
Ancestral state reconstructions. Ancestral state reconstructions of larval host substrate and secondary sexual characters in the AMC. Ancestral range reconstruction estimated under a DEC model and the most likely range is plotted on each node. Pie charts on nodes indicate probability of each state in same order as they are presented to the right of the phylogeny.

The restricted and unrestricted migration models run in the Lagrange analyses produced identical results at all nodes of interest. These results suggest that the AMC clade, as well as several component lineages with this group (*e.g.,* the *split tarsus* and *antopocerus* clades), originated in the islands of Maui Nui (Fig. 4). The ancestral range of the *spoon tarsus* subgroup was identified as Maui Nui or Hawaii.

The Hawaiian Islands are arranged in chronological order, with the oldest islands in the northwest, becoming progressively younger to the southeast. This has led to a general pattern in Hawaiian biogeography known as the progression rule [40], [41]. As new islands form, taxa from older neighboring islands can colonize them, leading to clades that have diversified “down” the chain. Basally branching lineages are found on older islands and more recently derived clades are found on younger islands. There were few clades of AMC species that followed a pattern of diversification predicted by the progression rule, contrary to what is displayed by many lineages in the Hawaiian Islands [4], [14], [27], [42], [66]. There was a Hawaiian Island clade of *antopocerus* nested within a Maui Nui clade. The Hawaiian spoon tarsus species were reconstructed as sister to the Maui Nui species. The clades of *ciliated tarsus* endemic to Kauai displayed extensive radiation and were derived from within lineages that are strongly supported as originating on Maui or Hawaii, the reverse from what is expected from the progression rule. These reconstructions are in conflict with the divergence dates we have estimated and we attempt to reconcile the findings below.

There is little variation in the ecological breadth of AMC species (Fig. 4). The majority of species with known oviposition and larval substrate utilize leaves from plants in the genus *Cheirodendron* in the family Araliaceae. The inferred ancestral state for every species group was reconstructed as larval utilization of Araliaceae leaves as a substrate (PP≥85%). The next most commonly used family of plants is Aquifoliaceae, but the transition to this host was inferred to have occurred only rarely – in our analyses all three times in the *hirsute tarsus* clade. There were at least two more transitions to Aquifoliaceae in the *antopocerus* species group (*antopocerus* species *D. cognata* and *D. entricocnema*, which belong to different complexes within the group, are not included in this phylogeny). The switch to utilizing Aquifoliaceae characterizes sister clades in the *spoon tarsus* subgroup, and predates the colonization of the Island of Hawaii. Transitions to other host substrates have occurred sporadically.

The majority of species groups diagnosed by a secondary sexual character were reconstructed as monophyletic and had ancestral nodes reconstructed with a high probability as having the same male secondary character morphology that defines the group (long antennae on *antopocerus*, spoons on the *spoon tarsus* and an apical lobe on the basitarsus of the *split tarsus* coincided with a PP>99%) (Fig. 4). However, the reconstructions identified a very low likelihood for any of these traits being present in ancestral AMC nodes (PP<1%), and none that correlated with the emergence of the *hirsute tarsus* clade. Likewise, the basitarsus to setae ratio showed little signal with the *hirsute tarsus* clade reconstructed as most having the most common ratio found throughout the AMC.

## Discussion

### AMC systematics

Our phylogeny is an improvement over recent studies of Hawaiian *Drosophila* phylogenetics that featured representatives of the AMC clade [7], [16], [67], due to this study's increased taxonomic and molecular sampling. We have resolved the relationships between the major species groups of the AMC with more resolution and statistical support than any molecular analysis to date (Fig. 1A and B, and Fig. 2). The relationships between the groups differ slightly from morphological predictions (Fig. 1A). This is the first analysis that is able to infer with strong support that the *antopocerus* species group is sister to the rest of the AMC, and that the *split tarsus* subgroup is sister to the hirsute tarsus clade. Additionally our study finds the *antopocerus* species group, *split tarsus* and *spoon tarsus* subgroups are monophyletic, in agreement with previous molecular [7], [38], [39], [45] and morphological hypotheses [28]–[31]. While the *ciliated tarsus* and *bristle tarsus* subgroups are morphologically distinguishable [29], [33] these analyses support the idea these subgroups are close relatives [7], [39] and are unable to recover these them as reciprocally monophyletic. We predict this lack of resolution between the two subgroups is the result of rapid divergences at the base of the *hirsute tarsus* clade that could not be resolved, and the diversification rate analyses corroborate this. While it is possible that hybridization and incomplete lineage sorting have caused conflict between the unlinked loci, examination of individual genealogies suggests that the poor support at the base of the *hirsute tarsus* clade is due to an apparent lack of phylogenetically informative characters (Fig S1–S11). Within each species group we infer relationships that are congruent with many of the previous molecular and morphological hypotheses [7], [28]–[30], [45].

### Divergence dating and Biogeography of the AMC

The Hawaiian Drosophilidae (Hawaiian *Drosophila* and *Scaptomyza*) are hypothesized to have descended from a single colonization event on the Hawaiian Archipelago and quickly diverged and radiated [16], [33], [68]–[71]. Divergence times within the Hawaiian *Drosophila* have been subsequently dated under a variety of assumptions [10], [15], [16], [67], [72]. Using a combination of biogeographic and external calibrations, we inferred similar dates of divergence as [10] and [16] and some dates inferred by [67]. Different calibrations and taxa were used in all three studies, which may have led to this discrepancy. While any molecular divergence dating should be interpreted with caution we are confident that due to the use of well known island ages [58], external calibration points from other Drosophilidae studies, and an expanded sampling of AMC taxa, the dates in this study provide the best estimates of a timeline of diversification within the AMC to date. We estimate that the extant lineages of Hawaiian *Drosophila* started diversifying about nine mya (95% HPD 6.57/11.82 mya) (Fig. 2). This is a period of high topographic diversity, when the islands Gardner and Necker Islands, now both nearly submerged, were both large in area with multiple islands between them [9]. Given that the other lineages diverged when Gardner and Necker were high, followed by a lack of lineage formation in the AMC until the emergence of the island of Kauai, we propose that diversification in the Hawaiian *Drosophila* may have been episodic, with species diversity associated with topographic diversity.

Two dated molecular phylogenies have included more than one AMC species [16], [67] and while [67] infers dates older than this study by four million years, [16] infers similar ages. The crown of the *antopocerus* species group diversified approximately 2.4 mya, at about the same time that the oldest islands of Maui Nui were in the midst of shield building [62]. This corresponds with the inferred ancestral range in Lagrange and corroborates earlier predictions of how the *antopocerus* species group diverged [28]. The *split tarsus* diverged from the rest of the *hirsute tarsus* subgroup about 3.5 mya, and both started diversifying about three mya, around the time of the formation of the island of Oahu. The youngest subgroup in the AMC, the *spoon tarsus*, started to diversify around 1.8 mya, likely on Maui Nui, and had since colonized and diversified on the Island of Hawaii. The Hawaii Island endemics in this group split into two lineages before the island of Hawaii was habitable (∼0.9 mya) and ancestral lineages on Maui may have since gone extinct.

The phylogeography of Hawaiian clades may recapitulate the progression rule, where older lineages are found on older islands and younger lineages on younger islands. This pattern is a paradigm in Hawaiian biogeography [27], [41], [73], but one that is not strictly observed [58], [74]. However, the ancestral range reconstructions do not reproduce this pattern in many lineages in the AMC (Fig. 2). Even though the timing of the AMC divergence is estimated as occurring on the older Hawaiian high islands (Kauai and Oahu), the ancestral range reconstruction identifies that most of the diversification occurred on the younger islands (Maui Nui and Hawaii). Similar to the *haleakalae* species group, we did not observe a pattern of progression down the island chains in many clades [7], [46]. This differs from the *planitibia* and other *picture wing* species groups where this pattern is clearly observed [22], [27].

Conflict between ancestral range reconstructions and dating of divergences is documented [66] and we interpret our results expecting the ancestral range reconstructions to inform patterns of diversification and biogeographic events extrapolated from our dating analyses. We hypothesize that a lack of a distinct biogeographic pattern in our range reconstructions may be the result of two processes. First, recent extinctions or incomplete species sampling on Kauai and Oahu are possible given the widespread habitat loss in recent and historic times, especially on Oahu. This is likely to have occurred on some level and will influence our reconstructions; in particular, the absence of the Oahu representative of the *antopocerus* species group and three of the four Oahu *split tarsus* species may affect the analyses. Alternatively, these results may be the product of ancient extinctions in basal taxa of each lineage following rapid diversification of the extant clades on Oahu and/or Maui Nui. This hypothesis is supported by the placement of the well-sampled Kauai AMC species as highly derived and comprised mainly of two clades, one of *bristle tarsus* species and one in the *split tarsus* subgroup (Fig. 4), indicating infrequent back-colonization from younger islands followed by within-island speciation. Likewise, all sampled Oahu species are derived within their respective clades. Maui Nui has been separated and connected multiple times in the islands approximately two million year history, most recently connected during the last glacial maximum [62]. This has alternately led to a high degree of topographic diversity and increased area, followed by periods of isolation between volcanic mountains, which are expected to promote speciation [75], [76]. A large proportion of the known AMC diversity is found on the islands of Hawaii and Maui Nui (79%) [29] and Maui Nui has been hypothesized to be a crucible of diversity in Hawaiian lineages [77]. Likely a combination of these factors has influenced our range reconstructions to some degree.

### Drivers of diversification

Since we were able to resolve the relationships with high support between many of the AMC species groups we can test hypotheses of what has led to the diversification between these groups for the first time. The difficulty in resolving the relationships between the *bristle* and *ciliated tarsus* subgroups, in spite of the use of multiple nuclear and mitochondrial markers, indicates that these taxa underwent a rapid radiation (Fig. 2), which is corroborated by our analysis. Most of the diversification within the AMC occurred recently, within the past ∼ 3 my, which would explain previous difficulty in inferring the relationships between the AMC lineages. Like other rapid radiations (e.g. [78]–[80]) Hawaiian *Drosophila* lineages are poorly resolved or lack support at the basal nodes despite increasing taxonomic and molecular sampling [7], [27], [38], [39], [46]. This rapid radiation may be due to an increase in speciation rate or a decrease in extinction rate. The change in the rate of speciation indicates that something in the environment or biology of the lineage changed. Based on previous studies on Hawaiian *Drosophila*, we predict three factors may have driven this diversification: (1) The *hirsute tarsus* subgroup may have expanded their host range to exploit a previously unused resources that allowed them to adaptively radiate across the Hawaiian Islands; (2) novel secondary sexual characters provide new substrates for sexual selection to drive divergence; (3) around the time the *hirsute tarsus* clade started to diversify there was an increase in available landscape – the current high islands were forming and increased topographic diversity has been shown to increase biodiversity in other Hawaiian lineages [4], [81]. This may be due to the availability of open niches reducing the extinction rate or an increased speciation rate as new niches become available. We consider each of these possibilities below.

The *picture wing*, *ateledrosophila* and *nudidrosophila* (PNA) clade utilizes several families of plants [7], [21] and diversification in this group may be linked to adaptation to different plants used as oviposition sites [23]. The diverse *modified mouthpart* clade also exploits a wide range of host plant families [7]. This expansion of resource use has helped to define the Hawaiian *Drosophila* as an adaptive radiation [65], but is not a universal trait of the entire clade. Indeed, we confirm that host use divergence is unlikely to have driven the diversification in the AMC clade since species in this group display a nearly uniform ecological lifestyle across the Hawaiian Islands: larvae from almost all species use the decaying leaves of species from the family Araliaceae as a substrate [21]. The transition to utilizing Aquifoliaceae leaves has occurred multiple times in the AMC, but does not appear to lead to increased diversity. This is in contrast with other diverse Hawaiian *Drosophila* clades and leads us to expect that the AMC may have diversified for other reasons.

Sexual selection has likely influenced speciation in the AMC. This is evidenced by the diversity of morphologies [28]–[31], behaviors [32], [34]–[37] and chemical signaling [82] that are associated with sexual selection and described in species of the AMC. Geographic isolation followed by a random and slight change in the way these secondary sexual characters are used could cause pre-mating isolation when sister species of Hawaiian *Drosophila* came back into contact with each other [83], [84] and secondary sexual characters have been shown to be important in mate choice [43]. The males of many AMC will vibrate and semaphore their wings in species distinctive patterns in the vicinity of conspecific females [32], [34]. Courtship song and cuticular hydrocarbons are diverse and stereotypical to species in the Hawaiian *Drosophila* and are likely used in identifying conspecific mates in the AMC [82], [85]–[87]. In at least the *antopocerus* group there are sophisticated structures for sensing these traits [88].

While many of these traits are very well characterized in a few AMC species, they are not known widely across the AMC lineage. This has led us to focus on the well defined eponymous morphological characters of each AMC species group, which are used in sexual displays and mating behaviors [29], [32]. We confirm these traits as being diagnostic for different species groups with high confidence for the first time (Figure 4). The *hirsute tarsus* clade is identifiable by a suite of different secondary sexual characters, but none that arose coincidently with the increase in diversification rate seen in this group. The ancestral node of the *hirsute tarsus* clade is reconstructed as having a bristle length to tarsus length most commonly found in all AMC groups, including those outside of the hirsute tarsus clade. We do not exclude the possibility of sexual selection driving an increase in the rate of diversification in the AMC, but also do not find support in the secondary sexual character data we explored. The evolution of complex secondary sexual characters can be rapid, especially in Hawaiian *Drosophila* [89], and in many other *Drosophila* species [86], [90], [91]. Future research characterizing the cuticular hydrocarbons and mating behaviors of these species could provide support for the importance of sexual selection in driving AMC speciation.

The AMC are inferred to have started diversifying around 4.40 mya (95% HPD 3.45/5.45 mya), which broadly corresponds with when Kauai would have been mature, and thus had wet forest habitat and topographic diversity, similar to Maui, but prior to Oahu and after other islands had largely sunk (i.e. it was the only big island). The later increase in rate of diversification associated with the hirsute tarsus clade is associated with the emergence of the island of Oahu 3.44 mya (95% HPD 2.72/4.22 mya). Increased area and topographic diversity in islands is expected to drive high levels of diversification [4], [75] and speciating down the island chain may have driven the AMC, and more specifically the *hirsute tarsus* clade's, diversity. While the oldest subgroup in the *modified tarsus* species group, the *split tarsus*, includes a large portion of species from Kauai, the oldest lineage in the AMC, the *antopocerus*, are found almost entirely on the islands of Maui Nui. The spatial heterogeneity this island group has recently experienced may also lead to AMC diversity. Given the timing of speciation events, the diversification of the AMC lineage may be the result of the emergence of a heterogeneous landscape that included Oahu and Maui Nui in addition to Kauai. This does not preclude other factors from influencing diversification rates within clades of AMC, and by identifying the dating of events such as the arrival of the genus *Cheirodendron* to the islands, and the mating behaviors of these species groups we can further test many of these hypotheses.

## Supporting Information

Figure S1 **Mitochodrial phylogeny.** Bayesian topology shown. Black dots at nodes indicate posterior probabilities > 0.9 and RAxML bootstrap values > 70.
(EPS)

Figure S2 ***Fz4*** **genealogy.** Bayesian topology shown. Black dots at nodes indicate posterior probabilities > 0.9 and RAxML bootstrap values > 70.
(EPS)

Figure S3 ***Snf*** **genealogy.** Bayesian topology shown. Black dots at nodes indicate posterior probabilities > 0.9 and RAxML bootstrap values > 70.
(EPS)

Figure S4 ***Yp1*** **genealogy.** Bayesian topology shown. Black dots at nodes indicate posterior probabilities > 0.9 and RAxML bootstrap values > 70.
(EPS)

Figure S5 ***Yp2*** **genealogy.** Bayesian topology shown. Black dots at nodes indicate posterior probabilities > 0.9 and RAxML bootstrap values > 70.
(EPS)

Figure S6 ***Kl-2*** **genealogy.** Bayesian topology shown. Black dots at nodes indicate posterior probabilities > 0.9 and RAxML bootstrap values > 70.
(EPS)

Figure S7 ***Boss*** **genealogy.** Bayesian topology shown. Black dots at nodes indicate posterior probabilities > 0.9 and RAxML bootstrap values > 70.
(EPS)

Figure S8 ***Ef1-g*** **genealogy.** Bayesian topology shown. Black dots at nodes indicate posterior probabilities > 0.9 and RAxML bootstrap values > 70.
(EPS)

Figure S9 ***Ntid*** **genealogy.** Bayesian topology shown. Black dots at nodes indicate posterior probabilities > 0.9 and RAxML bootstrap values > 70.
(EPS)

Figure S10 ***Pds5*** **genealogy.** Bayesian topology shown. Black dots at nodes indicate posterior probabilities > 0.9 and RAxML bootstrap values > 70.
(EPS)

Figure S11 ***Wee*** **genealogy.** Bayesian topology shown. Black dots at nodes indicate posterior probabilities > 0.9 and RAxML bootstrap values > 70.
(EPS)

Figure S12 **Maximum likelihood topology.** Values on nodes are bootstrap support.
(EPS)

Table S1 **List of individuals with mitochondrial sequences.** Barcode refers to O’Grady Lab bar-coding conventions and can be referred to identify full collection details. EM Barcode refers to voucher label associated with the sample provided by the Essig Museum of Entomology. Island refers to island where individual was collected. FB indicates sequences downloaded from FlyBase.
(XLSX)

Tables S2 **List of individuals with nuclear sequences.** Barcode refers to O’Grady Lab bar-coding conventions and can be referred to identify full collection details. FB indicates sequences downloaded from FlyBase. ST3 indicates which of the three sequences that are too short to be deposited onto GenBank (<200 bp) are included in Table S3.
(XLSX)

Table S3 **Bride of sevenless (** ***boss*** **) sequences too short to deposit onto GenBank.**
(XLSX)

Table S4 **Partitions and their associated substitution models.** Models listed here were used in Bayesian analyses.
(XLSX)
